# Postoperative Course and Supportive Care in Very Elderly Cecum Cancer Patients

**DOI:** 10.7759/cureus.61919

**Published:** 2024-06-07

**Authors:** Yojiro Ishikawa, Eisaburo Ishikawa, Kazuhiro Ishikawa

**Affiliations:** 1 Radiology, Tohoku Medical and Pharmaceutical University, Sendai, JPN; 2 Internal Medicine, Ishikawa Clinic, Hitachi, JPN; 3 Dentistry, Ishikawa Clinic, Hitachi, JPN

**Keywords:** palliative surgery, palliative radiation therapy, best supportive care, bsc, older patient

## Abstract

In developed countries like Japan, the size of the older population is rapidly increasing. Malignant neoplasms rank as the leading cause of death among the geriatric population of Japan, emphasizing the rising demand for cancer care in this demographic. Older patients, besides facing physical and cognitive challenges, are also affected by their social environment, necessitating tailored interventions. Few case reports have detailed the progress of cancer treatment in nonagenarian patients. This study presents the progress of two very old patients with cancer. The first case, a 95-year-old female with chronic constipation underwent emergency surgery for a cecal tumor. Despite initial improvements, her mobility declined after surgery, leading to institutionalization. Recurrent hospitalizations ensued with complications culminating in her death approximately 20 months after surgery. In the second case, a 94-year-old male, initially declining aggressive treatment for a suspected ileocecal malignancy, later opted for supportive care. Despite stable conditions, he eventually died at home after experiencing progressive weakness, which was approximately 20 months after the initial diagnosis.

These cases shed light on the management of elderly patients with ileocecal cancer, illustrating the divergent trajectories between surgical intervention and supportive care. The tumor did not recur in the patient who underwent surgery; however, the independence in performing daily living activities declined significantly. In the case managed with the best-supportive care, progression was slow; however, severe anemia became a concern toward the end of life.

## Introduction

Japan, a developed country, has a rapidly aging population, where 28.4% of the total population was 65 years or older in 2019, the highest in the world, and it is estimated to reach 38.4% by 2065 [1]. Malignant neoplasms are the leading cause of death among older people in Japan, and the need for medical care for older people with malignant tumors is increasing [2].

In addition to deteriorating physical function and cognitive problems, older people are more likely to be affected by the social environment, such as the family environment and community characteristics, and they may require a different response than the general adult population [3]. Despite reports indicating successful completion of standard treatment for malignant tumors in patients aged >80 years, many of them do not describe in detail the course after completion of treatment [4,5].

Cases with complete standard treatment are rare, and most reported cases are those that received the best supportive care (BSC) [6]. This study summarizes the progress of malignant tumors in nonagenarian patients (approximately 95 years old) with and without BSC from the perspective of palliative care and primary care.

## Case presentation

Case 1

A 95-year-old female experienced chronic constipation for several years and was regularly taking magnesium oxide, lubiprostone, and Japanese Kampo medicine. She was able to walk and had an Eastern Cooperative Oncology Group (ECOG) performance status (PS) of one. Her activities of daily living (ADLs) were relatively well maintained; although her cognitive function was not formally assessed, it did not significantly interfere with her daily life. She frequently complained of abdominal tightness; however, no apparent abdominal abnormalities were found on computed tomography, and endoscopy was not pursued because of her advanced age.

**Figure 1:**
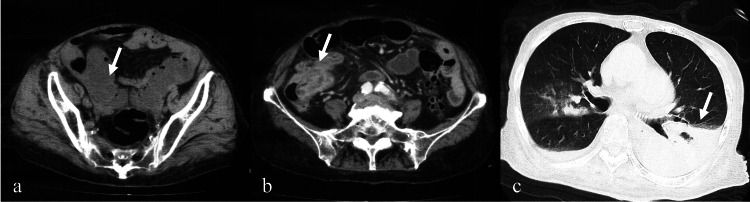
Abdominal and chest computed tomography of case 1. Abdominal computed tomography (CT) revealed small intestine dilation and intestinal effusion, indicating intestinal obstruction (a, arrow). Contrast-enhanced CT displayed a contrast-enhanced mass shadow from the terminal ileum to the proximal colon (b, arrow), with dilation of the intestinal tract. A chest CT performed 19 months after surgery revealed pneumonia and pleural effusion without apparent lung metastasis (c, arrow).

During a visit to a local general hospital for abdominal pain and nausea, a thorough examination revealed a mass lesion in the distal colon and ileum, accompanied by intestinal tract dilatation and effusion on the proximal side of the mass (Figure 1, panels a and b). A diagnosis of intestinal obstruction due to a tumor in the ileocecal region was established, leading to emergency surgery. After mass removal, the final diagnosis was cecal cancer pT2N0M0. Owing to her advanced age, further postoperative treatment or follow-up was deemed challenging.

**Figure 2:**
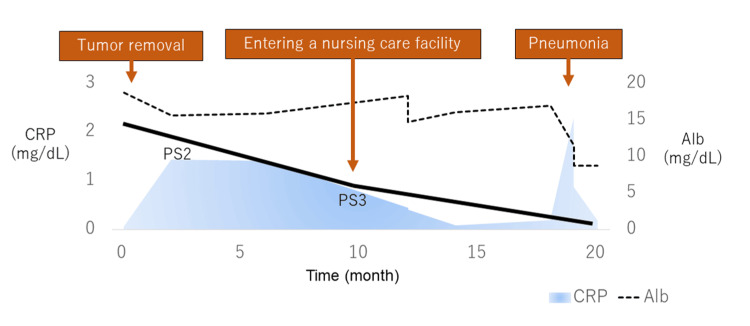
Timeline of the postoperative course of case 1. The patient’s performance status gradually deteriorated after the surgery, and approximately six months later, home-based care became impractical leading to admission to a nursing facility. The patient’s albumin levels gradually decreased, and the inflammatory markers remained persistently high reaching peak levels toward the end of the patient’s life. Alb: albumin; CRP: C-reactive protein; PS2: performance status of 2; PS3: performance status of 3

Two months after surgery, the patient continued treatment under the care of a local physician, resulting in an improvement in chronic constipation. However, her mobility declined after surgery, transitioning from walking independently to requiring a wheelchair, and her ADLs deteriorated. Although her postoperative recovery was favorable, six months later, she could not tolerate home care and was admitted to a facility. Approximately one year after surgery, she had recurrent hospitalizations for urinary tract infections and pneumonia. In addition, she had pressure ulcers on her buttocks and cellulitis, and she gradually required full assistance with eating and defecation. Despite her cognitive impairment, she could often express her wishes. At one year and eight months after surgery, she died because of worsening pneumonia and pleural effusion (Figure 1, panel c). No evidence of recurrent metastasis of appendiceal cancer was observed (Figure 2).

Case 2

A 94-year-old male, who was typically in good health, was taking medications for high blood pressure and diabetes management. He had a history of prostate hypertrophy but was under surveillance without active treatment. He had no indications of heart, liver, or kidney dysfunction. Despite not living alone, he maintained independence in performing ADLs, engaged in fieldwork and walks during the day, and regularly socialized at a pub in the evenings. He had never smoked.

**Figure 3:**
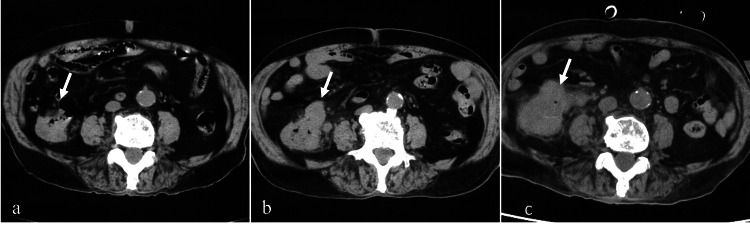
Abdominal computed tomography of case 2. Abdominal computed tomography scans at various time points are as follows: at the time of diagnosis (a), six months (b), and 18 months (c). The tumor from the terminal ileum to the proximal ascending colon shows progressive enlargement over time (arrows).

**Figure 4:**
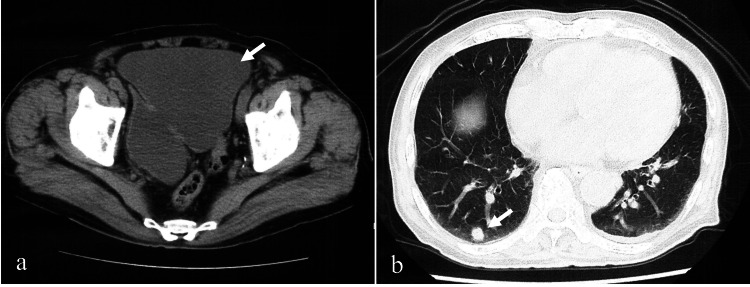
Pelvic and chest computed tomography of case 2. Abdominal computed tomography revealed chronic bladder retention and urinary retention throughout the disease course (a, arrow), along with the presence of a bladder diverticulum. Chest CT taken at 18 months into the disease course shows a solitary lung metastasis without associated symptoms (b, arrow).

He experienced constipation for several months, for which he was initially prescribed laxatives at a clinic, which were ineffective. Because of his reluctance to pursue aggressive treatment, he delayed seeking medical attention, despite suspicion of malignancy. Approximately six months later, a thorough evaluation at a general hospital revealed a mass lesion in the ileocecal region and a biopsy confirmed adenocarcinoma (Figure 3, panels a-c). Although surgical and anticancer options were offered upon hospitalization, the patient declined aggressive treatment and was discharged for observation without intervention. He maintained the ability to walk, with an ECOG PS of 1. For six months following discharge, his condition remained stable despite urinary difficulties and retention because of prostate hypertrophy, necessitating urethral catheterization (Figure 4, panel a).

**Figure 5:**
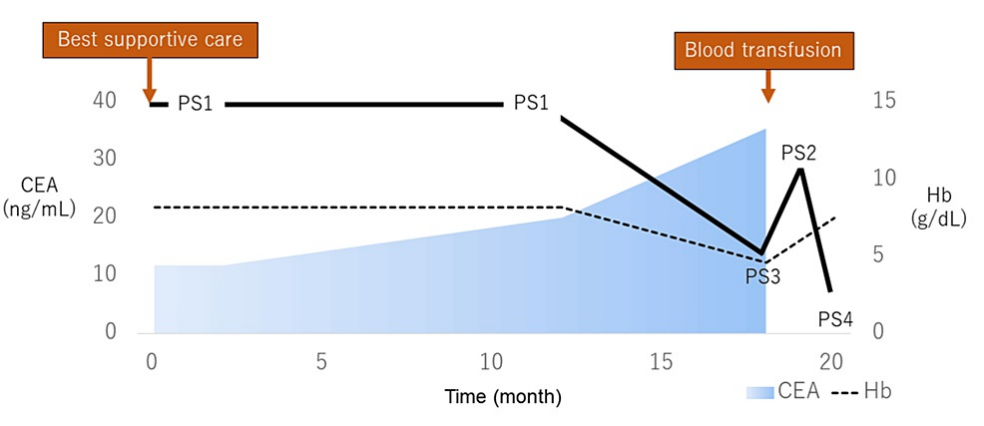
Timeline of the postoperative course of case 2. The symptoms progressed relatively slowly, with a relatively preserved performance status. Anemia progressed gradually, with a sudden drop in hemoglobin (Hb) levels noted at 18 months because of hemorrhage. Despite a decline in performance status, a blood transfusion effectively improved the patient’s anemia and performance status. However, shortly thereafter, the patient’s overall condition deteriorated rapidly, leading to her death at home. CEA: carcinoembryonic antigen; Hb: hemoglobin; PS1: performance status of 1; PS2: performance status of 2; PS3: performance status of 3; PS4: performance status of 4

Nine months after discharge, he presented with tumor-related bleeding, prompting consideration of radiotherapy. However, due to travel inconvenience (requiring a 30-min drive from home), he preferred treatment at a local clinic or hospital within walking distance. With progressing anemia (hemoglobin {Hb} 4.4 g/dL), he was hospitalized for a transfusion of four units of red blood cells, resulting in Hb recovery to 8.8 g/dL and rapid symptom improvement, transitioning to outpatient care. Progression other than local disease was slow, with only asymptomatic pulmonary metastases (Figure 4, panel b). He continued his recuperation with home visits and supportive therapy from his local doctor. Over time, symptoms worsened, marked by progressive weakness and immobility, leading to his death at home (Figure 5).

## Discussion

Although the incidence of malignant tumors in elderly people is believed to vary depending on the aging status of each region, the incidence of digestive tract cancers, such as colorectal cancer, among those aged >80 years is more than 20% [7]. The percentage of patients aged ≥80 years among patients with esophageal cancer was reported to range from 4.4% to 5% [8]. With the aging population, the proportion of elderly patients with gastrointestinal malignancies is expected to further increase. In Japan, life expectancy in the absence of cancer at 95 years of age is 2.4 years for males and 3.0 years for females and is expected to rise [9]. All patients in this study survived approximately 18 months from disease onset; however, whether cancer treatment affected life expectancy was difficult to assess because a survival rate can be considered equivalent to expected life expectancy. The association between chronic constipation and colorectal cancer is frequently debated, and early detection is advocated. However, at 95 years of age, screening via gastroscopy or colonoscopy is deemed challenging [10]. Given the anticipated life expectancy, the extent to which early detection might affect the prognosis remains uncertain.

In a previous report, BSC was recommended in 50% of patients aged >85 years with cancer spreading from the cecum to the distal transverse colon [11]. Reasons cited for opting for BSC in very old people include poor PS, advanced age, and advanced dementia, suggesting that organ function decline may be less problematic in fewer cases [12]. In patients with advanced dementia, comprehension and cooperation may be challenging, and postoperative pain and restrictions may not serve their best interests.

A comprehensive surgical suitability assessment to determine whether a patient is suitable for invasive treatment objectively is often challenging, particularly when dealing with older people who may require urgent interventions. The dearth of substantial evidence for treating old people has led many to rely on clinicians’ experience to make decisions [13,14].

Both cases presented involved lesions of the cecum to the proximal transverse colon and had a relatively favorable prognosis. In case 1, the patient underwent urgent surgery for ileus symptoms. In previous reports, older patients were frequently managed urgently. Those who underwent surgery and radiation therapy with cancer spreading from the cecum to the proximal transverse colon exhibited a better prognosis than those with involvement of the distal transverse or sigmoid colon [11]. Moreover, there is a postoperative decline in physical strength, walking difficulty, and deterioration in PS, emphasizing the importance of a thorough preoperative evaluation [15].

Conversely, when BSC is chosen for patients with relatively stable PS, support from the surrounding community becomes essential. Advanced age and deteriorating patient health often lead to a diminished quality of life for the patient’s family and caregivers [16]. In case 2, the patient appeared to have a relatively stable clinical condition. Although gastrointestinal bleeding is a common symptom of gastrointestinal cancer, in case 2, the patient’s symptoms improved, and quality of life improved after a blood transfusion. Anemia and blood transfusion related to rectal cancer could affect prognosis [17]. Radiation therapy is regarded as a viable option for hemostasis in such cases of hemorrhage [18]; however, one of the challenges for very old persons is access to social support, including facilities offering radiation therapy [19].

Chemotherapy was not initiated because of the patient's advanced age. However, cases of cancer in old patients being locally controlled with chemoradiotherapy have been reported [12,20]. Despite reports of successful chemotherapy in very old patients, concerns over radiation exposure to caregivers because of self-removal of intravenous needles or urination are prevalent, along with the effect on medical personnel. Patients may also require physical restraints, exacerbating their suffering [21].

## Conclusions

This report of two cases focuses on the course of elderly patients, approximately 95 years of age, with ileocecal cancer, highlighting the differences in the course between those who underwent surgical intervention and those managed with BSC. Collecting or randomizing numerous cases involving individuals approximately 95 years of age is expected to be exceedingly difficult, underscoring the significance of this study. Although both patients initially were thought to have a short life expectancy, they survived for approximately two years. The patient who underwent prompt surgery because of ileus symptoms remained free of cancer but experienced a significant decline in ADLs, necessitating institutional care. In the case managed with the BSC, progression was slow; however, the patient’s condition deteriorated rapidly in the terminal stage. Discussing the standard of care for similar cases is challenging given the limited scope of this report of only two cases; however, this report will offer valuable insights to clinicians encountering similar scenarios.
